# Ethanol Extract of *Oldenlandia diffusa* Herba Attenuates Scopolamine-Induced Cognitive Impairments in Mice via Activation of BDNF, P-CREB and Inhibition of Acetylcholinesterase

**DOI:** 10.3390/ijms19020363

**Published:** 2018-01-25

**Authors:** Jung Eun Lee, Hyo-Sook Song, Moon Nyeo Park, Sung-Hoon Kim, Bum-Sang Shim, Bonglee Kim

**Affiliations:** 1 Department of Pathology, College of Korean Medicine, Graduate School, Kyung Hee University, 1 Hoegi-dong, Dongdaemun-gu, Seoul 130-701, Korea; jungeunlee@khu.ac.kr (J.E.L.); mnpark@khu.ac.kr (M.N.P.); sungkim7@khu.ac.kr (S.H.K.); 2 Department of Science in Korean Medicine, College of Korean Medicine, Graduate School, Kyung Hee University, 1 Hoegi-dong, Dongdaemun-gu, Seoul 130-701, Korea; shs331@khu.ac.kr

**Keywords:** *Oldenlandia diffusa* Herba, acetylcholinesterase, scopolamine, brain-derived neurotrophic factor, phospho-cAMP response element-binding protein

## Abstract

Though *Oldenlandia diffusa* Herba (ODH) has been known to exhibit anti-cancer and anti-inflammatory effects, its anti-amnestic effect has never been reported so far. The aim of this present study was to elucidate the anti-amnestic effect of ODH. ODH pretreatment significantly reduced escape latency of scopolamine treated Institute of Cancer Research (ICR) mice compared to untreated control groups in a Morris water maze test. Similarly, the passive avoidance test showed that ODH treatment recovered the scopolamine induced amnesia in the ICR mouse model. Concentration of Ach in brains of ODH treated mice was increased compared to that of scopolamine treated mice. In addition, activity of acetylcholinesterase (AChE) was notably decreased by ODH. The protein expression of brain-derived neurotrophic factor (BDNF) and phospho-cAMP response element-binding protein (p-CREB) (Ser133) was increased in ODH pretreated group compared to control group. Consistently, immunohistochemistry (IHC) revealed the elevated expression of brain-derived neurotrophic factor (BDNF) and p-CREB in brains of ODH treated mice compared to the control group. Overall, these findings suggest that ODH has anti-amnestic potential via activation of BDNF and p-CREB and inhibition of AChE in mice with scopolamine induced amnesia.

## 1. Introduction

Alzheimer’s disease (AD), a major neurodegenerative disorder, is characterized by a progressive loss of cognitive abilities, which affects memory and learning dysfunction [1]. According to World Alzheimer Reports, 46.8 million people have been suffering from AD in 2016 worldwide [2]. The number of AD incidence is increasing over the next two decades, and 1 in 85 people will be diagnosed with AD by the year 2050 [3,4]. Nonetheless, there is neither an effective therapy, nor a vaccine for AD to date [5]. 

Ach, a neuromodulator, is reported to have a crucial role in the process of perception, attention, learning and memory [6]. In brief, Ach releases in the hippocampus and brain cortex, which is involved in memory and learning [7]. High Ach concentration increases the magnitude of afferent input through action of nicotinic receptors in the cortex [8]. On the other hand, low concentration of Ach was seen in aging and AD, associated with brain cholinergic deficits [9]. 

AChE, an enzyme, degrades Ach by catalyzing the degradation of it and other choline esters. Low level of AChE activity, elevated concentration of Ach in the hippocampus and brain cortex [10]. Dysfunction of the cholinergic system can trigger the defects of memory and learning processes, and targeting AChE activity is a promising strategy in AD or memory enhancing research. In fact, AChE inhibitors were approved for cognitive disorders and are the most extensive in all anti-dementia drugs [9,11].

Brain-derived neurotrophic factor (BDNF) is a growth factor in nervous system, located in cortex, hippocampus, etc. [12]. BDNF is transported from the entorhinal cortex to the hippocampus and associated with memory ability [13]. The low level of BDNF was detected in AD patients [14]. Interestingly, administration of BDNF to the entorhinal cortex of an amyloid precursor protein transgenic mouse line increased memory and learning skills and ameliorated age-related neural dysfunction in rodents [15]. BDNF strongly induces phosphorylation of cAMP response element-binding protein (CREB) at the site of Ser-133 and p-CREB interacts with other related proteins, involved in regulation of emotion and memory [16]. The CREB is a key transcription factor, regulating synaptic and neural plasticity related genes [17]. According to We et al., the expression of BDNF is decreased in the AD rat model [18]. 

Recently, anti-amnestic activity has been reported from natural products such as pine needle extract [19], *Avena sativa* extract [20] and *Actinidia argute* extract [21]. The extract of *Oldenlandia diffusa* is reported to have anti-inflammatory [22] and anti-cancer activity in colorectal [23], liver [24] and breast cancers [25]. Furthermore, its compounds such as ursolic acid and oleanolic acid were reported for their anti-amnestic and neuroprotective effects [26,27]. However, anti-amnestic activity of *Oldenlandia diffusa* Herba has never been revealed yet. Thus, in the present study, the anti-amnestic mechanism of ODH was examined in mice scopolamine induced cognitive impairment. 

## 2. Results

### 2.1. ODH Treatment Did Not Significantly Affect Body and Brain Weight Loss on ICR Mice

**Figure 1:**
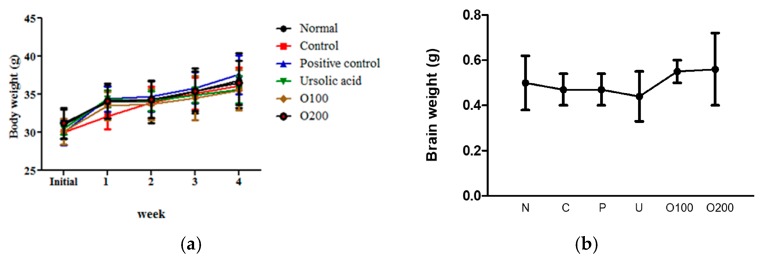
Body and brain weights of mice. (**a**) body weight of mice during the experimental period (28 days). Body weight was measured every week for four consecutive weeks. (*n* = 10 per group); (**b**) brain weights of mice four weeks after *Oldenlandia diffusa* Herba (ODH) treatment. After 12 h starvation, brains were removed. The brains were washed with normal saline, and dried. N: normal group, C: control group, P: tacrine treated positive control groups, U: ursolic acid treated groups, O100: ODH 100 mg/kg treated group, and O200: ODH 200 mg/kg treated group.

To evaluate the adverse effect of ODH, weights of ICR mice were measured every week during the diet period. As shown in Figure 1a, no significant weight differences were observed between normal group and any drug treated groups. Similarly, brain weights of ODH treated mice were not less than that of normal mice (Figure 1b). 

### 2.2. ODH Treatment Increased Learning and Memory Ability of ICR Mice

**Figure 2:**
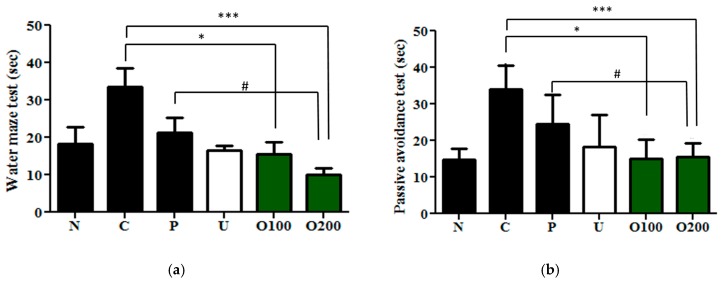
Anti-amnesic effects of ODH in mice with scopolamine induced amnesia. Mice were orally preadministrated with tacrine (2 mg/kg), ursolic acid (2 mg/kg), ODH (100 or 200 mg/kg) for four weeks. Mice were injected with scopolamine (3 mg/kg) to induce amnesia except for a normal group. (**a**) Effect of ODH on escape latency by Morris water maze test. Values are expressed as the means ± SD. * *p* < 0.05, *** *p* < 0.001 vs. scopolamine treated control group. # *p* < 0.05 vs. positive control group; (**b**) effect of ODH on scopolamine-induced memory deficit by the passive avoidance test. Values are expressed as the means ± SD. * *p* < 0.05 vs. scopolamine treated control group. *** *p* < 0.001 vs. scopolamine treated control group. # *p* < 0.05 vs. positive control group. N: normal group, C: control group, P: tacrine treated positive control groups, U: ursolic acid treated groups, O100: ODH 100 mg/kg treated group, and O200: ODH 200 mg/kg treated group.

Next, the behavioral memory improvement of ODH in scopolamine induced memory impaired mice was examined by using a Morris water maze test and passive avoidance test. As shown in Figure 2a, only the scopolamine injected control group took a longer time to find the platform throughout the three days of tests compared to the normal group. However, ODH treated groups significantly shortened the mean latency to find a platform compared control group (F_5.1197_ = 0.1724, O100: *p* = 0.0477; O200: *p* < 0.001). Furthermore, ODH200 groups showed a greater effect than the positive control, the tacrine treated group. In addition, a passive avoidance test was also performed with ODH treated mice. Consistent with Morris water maze test results, scopolamine induced memory deficits were notably protected by ODH treatment in passive avoidance tests. The effect of ODH (100 and 200 mg/kg) was significantly better than that of tacrine (2 mg/kg) (F_5.1197_ = 0.1724, *p* = 0.0336, Figure 2b). 

### 2.3. ODH Regulated Ach Concentration and AChE Activity in Scopolamine Treated Murine Brain

**Figure 3:**
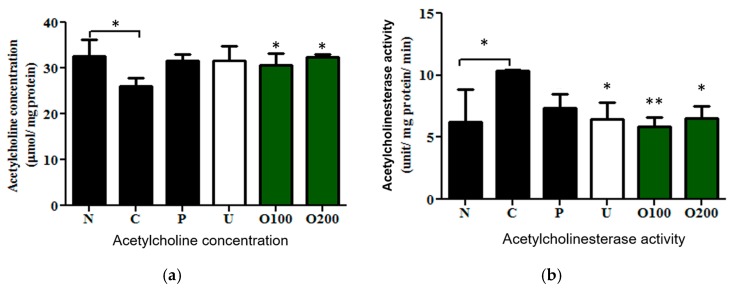
Effects of ODH on the Ach concentration and AChE activity in murine brains of scopolamine treated mice. Mice were orally preadministrated with tacrine (2 mg/kg), ursolic acid (2 mg/kg), ODH (100 or 200 mg/kg) for four weeks and injected with scopolamine (3 mg/kg) to induce amnesia. (**a**) evaluation of Ach concentration in brains of mice; (**b**) measurement of AChE activity in murine brains. Values are expressed as the means ± SD. * *p* < 0.05, ** *p* < 0.001 vs. scopolamine treated control group. N: normal group, C: control group, P: tacrine treated positive control groups, U: ursolic acid treated groups, O100: ODH 100 mg/kg treated group, O200: ODH 200 mg/kg treated group.

To verify whether ODH treatment regulates Ach or AChE in the brain tissue of mice, concentration of Ach and activity of AChE were measured. Scopolamine markedly reduced Ach concentration in the brain of mice, while ODH significantly increased Ach concentration (F_3.4921_ = 4.6493, Normal: *p* = 0.027; O100: *p* = 0.0123; O200: *p* = 0.0444, Figure 3a), which was similar to tacrine or ursolic acid treatment. Conversely, elevated activity of AChE by scopolamine injection returned to a normal level by the ODH treatment (F_4.2778_ = 75.6, Normal: *p* = 0.01357; ursolic acid: *p* = 0.03569; O100: *p* = 0.00314, O200: *p* = 0.03051, Figure 3b). 

### 2.4. ODH Increased the Expression of BDNF and Phosphorylated CREB Protein Expression in Mice

**Figure 4:**
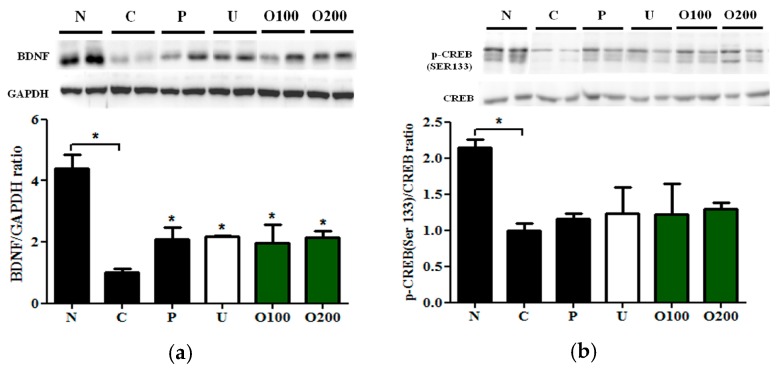
Effects of ODH on BDNF and CREB expression in murine brains. Mice were orally preadministrated with tacrine (2 mg/kg), ursolic acid (2 mg/kg), ODH (100 or 200 mg/kg) once a day for four consecutive weeks and injected with scopolamine (3 mg/kg) to induce amnesia. (**a**) Western blot analysis of BDNF and GAPDH in murine brains. Bar graphs represent the quantification of ratio of BDNF/GAPDH (brain-derived neurotrophic factor/Glyceraldehyde 3-phosphate dehydrogenase) protein expression; (**b**) Western blot analysis of p-CREB and CREB in murine brains. Values are expressed as the means ± SD. * *p* < 0.05 vs. scopolamine treated control group. Bar graphs represent the quantification of ratio of p-CREB/CREB protein expression. N: normal group, C: control group, P: tacrine treated positive control groups, U: ursolic acid treated groups, O100: ODH 100 mg/kg treated group, O200: ODH 200 mg/kg treated group.

**Figure 5:**
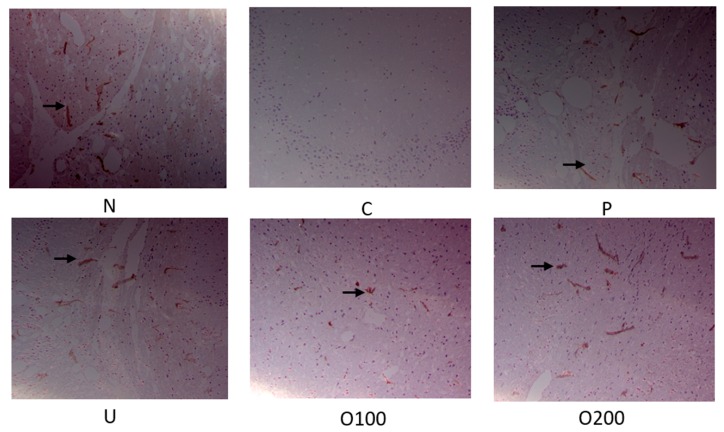
Immunohistochemistry staining of BDNF in murine brains. Mice were orally preadministrated with tacrine (2 mg/kg), ursolic acid (2 mg/kg), ODH (100 or 200 mg/kg) for four weeks and injected with scopolamine (3 mg/kg) to induce amnesia. Dissected brain was cut into coronal frozen sections (4 μm) and stained with BDNF antibody. The sections of hippocampus region were photographed at ×100 magnification. BDNF expression was observed in the hippocampus of mice (arrows). N: normal group, C: control group, P: tacrine treated positive control groups, U: ursolic acid treated groups, O100: ODH 100 mg/kg treated group, O200: ODH 200 mg/kg treated group.

**Figure 6:**
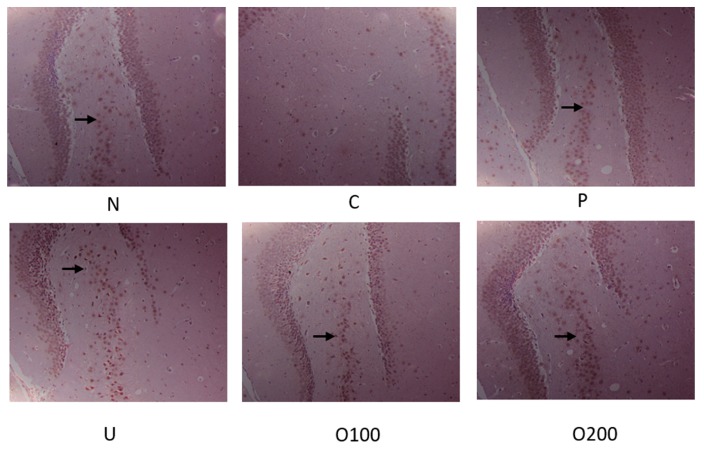
Immunohistochemistry staining of p-CREB in murine brains. Mice were orally preadministrated with tacrine (2 mg/kg), ursolic acid (2 mg/kg), ODH (100 or 200 mg/kg) for four weeks and injected with scopolamine (3 mg/kg) to induce amnesia. Dissected brain was cut into coronal frozen sections (4 μm) and stained with BDNF antibody. The sections of hippocampus region were photographed at ×100 magnification. The expression of p-CREB was observed in hippocampus of mice (arrows). N: normal group, C: control group, P: tacrine treated positive control groups, U: ursolic acid treated groups, O100: ODH 100 mg/kg treated group, O200: ODH 200 mg/kg treated group.

To elucidate which signal pathway was regulated by ODH treatment, Western blot analysis and immunohistochemistry (IHC) were conducted in dissected hippocampus of brains. As shown in Figure 4a, BDNF protein expression was reduced by scopolamine injection, which was recovered by ODH treatment. In addition, ODH treatment recovered decreased p-CREB (S133) expression by scopolamine (Figure 4b). The protein expression of BDNF and p-CREB in brains of mice were examined with IHC to confirm the results. Similar to Western blot analysis, the expression of BDNF and p-CREB was increased in ODH treated group, while that of BDNF and p-CREB was reduced by scopolamine injection (Figure 5 and Figure 6). 

## 3. Discussion

AD is a progressive disorder characterized by cognitive impairment by neuronal loss [28]. Recently, interests of delaying the process or improving symptoms of AD have been growing and growing [29]. The clinical symptoms of AD include short-term memory loss, delusions, apathy, depression, lateralization, language disorder, etc. [30]. Many researchers have used stem cell therapy [31], vaccine [32], and AChEIs [33] for AD therapy. However, there is no effective treatment available to cure this dreadful disease. Though recent some extracts/compounds of plants were reported to anti-amnestic efficacies [34,35], effective anti-amnestic agents have not been discovered yet.

Since scopolamine is known to show rapid anti-depressant effects in humans [36], and impair short-term memory and learning ability, it has been used for the Alzheimer’s disease animal model [37]. The present study revealed that pretreatment of ODH for 28 days protected the scopolamine induced amnesia ICR mouse model. According to Liang et al., ODH contains 2.363 ± 0.497 mg/g of ursolic acid (0.236%) and 0.514 ± 0.119 mg/g of oleanolic acid (0.0119%) [38]. The oleanolic acid showed neuroprotective effect [26]; however, ursolic acid is about 20 times rich in ODH and is reported for its AChE inhibitory effect [27], ursolic acid was used for positive control. The dosage of ursolic acid used was 2 mg/kg and that of ODH was 100 or 200 mg/kg. The ursolic acid in 100 or 200 mg of ODH is approximately 0.2363 or 0.4726 mg, respectively. In a Morris water maze test, ODH reduced the latency time to find the platform compared to control group, indicating that ODH ameliorated the amnesia induced by scopolamine injection. Lu et al. reported that treatment of ursolic acid (10 mg/kg/day) for 20 weeks reduced mean latency to half [39]. However, 100 mg/kg/day of ODH treatment lessened the mean latency to half, which contains only 0.2363 mg/kg of ursolic acid as mentioned. Likewise, scopolamine induced memory deficits were protected by ODH pretreatment in a passive avoidance test with scopolamine treated ICR mice. Pretreatment with a dose of 100 or 200 mg/kg of ODH for 28 days significantly reversed memory impairment induced by scopolamine injection, indicating that ODH recovers the spatial memory and learning ability in dementia mice. 

Ach, a major neurotransmitter in the central cholinergic system, is reported to have a role in learning and memory and is hydrolyzed by AChE [8]. The concentration of Ach and activity of AChE were investigated to evaluate the effect of ODH on memory loss in scopolamine treated mice. The pretreatment of ODH notably recovered the decreased concentration of Ach and increased AChE activity by scopolamine. ODH (100 mg/kg), which contains only 0.2363 mg of ursolic acid, showed almost the same effects of ursolic acid (2 mg) on AChE inhibition, which means ODH is almost nine times more effective than ursolic acid itself. Lu et al. elucidated that ursolic acid attenuated a high fat diet induced ER stress and cognitive defects in mice [39]. The anti-amnestic effect of ODH might be related to ER stress inhibition, and further mechanism study should be conducted.

It is well documented that BDNF is closely associated with learning and memory processes and CREB activation is required for BDNF expression in hippocampal neurons [40,41]. To examine the effect of ODH on BDNF and p-CREB expression, Western blot analysis and IHC were performed. Decreased expression of BDNF by scopolamine injection was significantly elevated by ODH pretreatment. Similarly, phosphorylation of CREB (S133) was recovered by OHD pretreatment by Western blot analysis and IHC. The elevated expression of p-CREB by ODH had no significance because of the error; however, IHC results confirmed the tendency. These results strongly suggest that ODH could be a safe and effective pharmaceutical for AD via modulation of Ach/AChE and CREB/BDNF signaling pathways. However, further studies on pharmacodynamics and pharmacokinetic of ODH are needed.

## 4. Materials and Methods

### 4.1. Chemicals and Reagents

Tacrine and ursolic acid were purchased from Sigma Aldrich (St. Louis, MO, USA). *Oldenlandia diffusa* Herba (200 g, dried) were harvested in Hongchungun, Gangwondo, Korea, and identified by Prof. Namin Baek from the Department of Oriental Medicine Biotechnology at the Kyung Hee University. A voucher specimen (No. KH-00725) was stored at the herbarium of Cancer Molecular Targeted Herbal Research Center of Kyung Hee University. The condition of the herbarium was as follows; humidity is between 45% and 55%, temperature levels is between 18 and 22 °C. The preparation and extraction were carried out as previously described [42,43]. Briefly, the ODH was extracted twice in 100% ethanol (EtOH, 1 L × 2) for 3 days each. The extracted solutions were filtered and evaporated to produce an EtOH extract (9.4 g, yield = 0.047). Antibodies for BDNF (Santa Cruz, CA, USA), p-CREB (Ser133), total-CREB (Santa Cruz, CA, USA) and GAPDH (Cell Signaling, Beverly, MA, USA) were purchased.

### 4.2. Animals

Male ICR mice (5 weeks old) were purchased from Samtako, BIOKOREA (Osan, Korea). The animals were acclimated for 7 days before the experiments. They were maintained under controlled temperature (22 ± 3 °C) and humidity (50 ± 10%) on a 12 h light/dark cycle (07:00~19:00). All of the animal experiments were approved by the Institutional Animal Care and Use committee (SEMI15-07, 3 January 2016), and the procedures followed were in accordance with the standards set forth in the eighth edition of Guide for the Care and Use of Laboratory Animals.

### 4.3. Drug Treatment 

**Figure 7:**
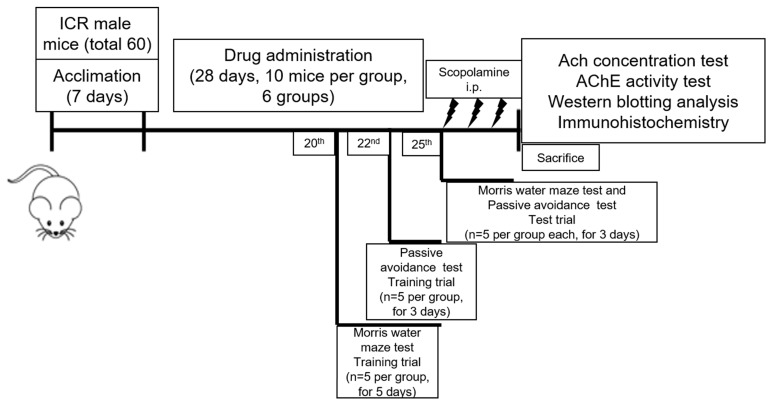
Timetable of the experiments.

After 1 week of acclimatization, mice were randomly assigned into 6 groups of 10 animals each: normal, control, tacrine (2 mg/kg), ursolic acid (2 mg/kg), ODH100 (100 mg/kg), ODH200 (200 mg/kg) (Table 1). The timetable of the experiments was shown in Figure 7. For normal and control groups, normal saline was orally given. Tacrine (9-Amino-1,2,3,4-tetrahydroacridine hydrochloride hydrate, Sigma Aldrich., St. Louis, MO, USA) is an AChE inhibitor, which was approved for AD, and ursolic acid (3β-Hydroxy-12-ursen-28-ic acid, Sigma Aldrich, St. Louis, MO, USA), a main compound of ODH, is reported as a potential therapeutic agent for AD. Thus, the two drugs were used as positive controls in this study [44,45]. The drugs were orally administrated daily for 28 days using intragastric (IG)-gavage. After 28 days of a diet period, scopolamine (Sigma Aldrich, St. Louis, MO, USA) (3 mg/kg) was injected intraperitoneally to control and ODH groups to induce memory impairment, while the same volume of saline was orally administered to normal group, 5 min prior to behavioral tests. Mice were starved for 12 h and sacrificed for experiments to avoid misleading biochemical parameters due to food.

### 4.4. Morris Water Maze Test 

The test was performed as previously described with minor modification [46]. In brief, a circular pool (100 cm in diameter, 35 cm in height) was filled with water (21~32 °C) at a depth of 15 cm. Mice were trained to find the hidden platform in the water maze for five consecutive days from the 20th day of administration of drugs (5 mice for each group, for 90–105 s per day). The mice were given for 90 s to find the hidden platform. When failed to find the platform in 90 s, mice were placed on it for 15 s. After 30 s, the escape latency (finding the submerged platform) was recorded. Acquisition trials were completed if all the mice found the platform within 90 s. A test trial was conducted on the 25th day using the same method of training trial for 3 days, and escape latency was recorded.

### 4.5. Passive Avoidance Test 

Passive avoidance test was conducted as described previously [47], using shuttle box (50 × 15 × 40 cm, electric grid floor, Ugo, Italy) that consisted of one illuminated (with lamp of 20 W) and one dark room (each 25 × 15 cm) and one connecting guillotine door (10 × 10 cm). The experiment was performed under quite environment (<60 dB). In 5 successive training trials, mice (5 mice for each group, for 90 s per each training trial) were placed in the illuminated room (1500 Lux) on the 22nd day of the drug administration. When mice moved to the dark room, an electric foot shock (3 mA) was given through the stainless grid for 3 s. After 24 h, the mice were placed in the light box while the connecting guillotine door was opened. A test trial was conducted on the 25th day using the same method of training trial for 3 days, and the latency to enter into the dark room was recorded for 5 min.

### 4.6. Tissue Preparation for Biochemical Analysis

The mice were decapitated under ether anesthesia after 12 h of starvation, and the brains were removed and weighed. Then, the brain was homogenized with homogenization buffer (12.5 mM sodium phosphate buffer pH 7.0, 400 mM sodium chloride). The tissue homogenates were centrifuged at 1000× *g* at 4 °C for 10 min, and the supernatants were used for biochemical analysis. 

### 4.7. Measurement of Acetylcholine Concentration

The concentration of Ach in the brain was measured according to a method previously described [48]. Briefly, 50 μL of brain homogenate was mixed with 1% of hydroxylamine 50 μL and FeCl_3_ (10% in 0.1 N HCl) 500μL. The absorbance (490 nm) was evaluated using a spectrophotometer.

### 4.8. Measurement of Acetylcholinesterase Activity

The activity of AChE, a marker for cholinergic neurotransmission, was determined according to a method described in previous studies with minor modifications [49] In brief, aliquots of homogenates of brain were measured by adding 0.1 M phosphate buffer (pH 8.0) 2.6 mL, 10 mM Ellman’s solution (10 mM 5,5′-dithio-bis-2-nitrobenzoic acid, 15 mM sodium bicarbonate) 100 μL, zymogen 0.2 mL, the change in absorbance was monitored at 405 nm for 2 min using a spectrophotometer. 

### 4.9. Western Blot Analysis

The brain tissues was added with PBS buffer (300 μL), homogenized with homogenizer and centrifuged 800 rpm, 10 s. The supernatant was lysed with lysis buffer 50 μL for 30 min in the ice and centrifuged at 14,000 rpm at 4 °C for 20 min. The protein concentrations were calculated using the Bradford assay [50]. The same amount of proteins were separated by electrophoresis on SDS-PAGE gel and electrotransferred onto a NC (nitro cellulose) membrane using a semi-dry transfer system (Bio-Rad, Hercules, CA, USA). After blocking with blocking buffer (0.5% skim milk, 1× PBST buffer) for 1 h and washing with 1× PBST buffer for 10 min (three times), the membrane was exposed to primary antibodies for BDNF (Santa Cruz, CA, USA), p-CREB (Ser133), total-CREB (Santa Cruz, CA, USA) and GAPDH (Cell signaling, Beverly, MA, USA). After 2 h, membrane was incubated with horseradish peroxidase (HRP)-conjugated secondary anti-mouse or rabbit antibodies (AbD Serotec, Kidlington, UK). The enhanced chemiluminescence (ECL) system (Amersham Pharmacia, Piscateway, NJ, USA) of Western Blot detection kit (Bio-Rad, Hercules, CA, USA) was used to visualize protein bands with Chemi-Doc (Bio-Rad, Hercules, CA, USA). 

### 4.10. Immunohistochemistry Staining Analysis

Dissected brain was wash and fixed with Bouin solution for 4 h. The brain was cryoprotected in 30% sucrose, embedded in tissue-freezing medium with liquid nitrogen, and cut into coronal frozen sections (4 μm). In addition, 10 μm tissue sections were deparaffined using heated Xylol and rehydration was conducted with subsequent lowered EtOH (100%, 80%, 50%, H_2_O), followed by treatment with blocking buffer (5% normal chicken serum in PBS and 0.3% Triton X-100 for overnight at 4 °C) and incubated with 1:100 diluted primary BDNF (Santa Cruz, CA, USA), p-CREB (ser133) (Santa Cruz, CA, USA) antibodies at 4 °C overnight. The expression of proteins was visualized using 3.3'-diaminobenzidine tetrahydrochloride (DAB). All immunoreactions were observed using an Axio vision 4.0 fluorescence microscope (Carl Zeiss, Upper Cohen, Germany).

### 4.11. Statistical Analysis

All data were expressed as the means ± SD. The statistical significance for Western blot analysis was determined by Student *t*-test for comparison of two groups. Two-way analysis of variance (ANOVA) with a post hoc test was used for Morris water maze test, passive avoidance test and Ach/AChE measurement to assess the significance of differences (groups × trial session (day)). *p <* 0.05 considered to be statistically significant difference. 

## 5. Conclusions

ODH pretreatment significantly increased memory and learning ability in ICR mice with scopolamine induced amnesia by Morris water maze test and passive avoidance test. In addition, ODH pretreatment increased concentration of Ach and reduced AChE activity compared to control group. Furthermore, the protein expression of BDNF and p-CREB were elevated in the ODH pretreated group compared to the control group by Western blotting and IHC. Overall, our findings support scientific evidences that ODH can be a potent anti-amnestic agent via activation of BDNF and p-CREB and inhibition of AChE. However, further in vitro or clinical studies are needed to verify the anti-amnestic effect of ODH in humans in the future. 

## Figures and Tables

**Table 1:** Experimental design.

Number	Group	Dose (mg/kg)	Design		Number
1	Normal		Saline		10
2	Control			Scopolamine 3 mg/kg i.p. ^1^	10
3	Positive control	2	tacrine.		10
4	Positive control	2	Ursolic acid		10
5	ODH	100	O100		10
6		200	O200		10

^1^ i.p. = Intraperitoneal injection.
