# Lee, J.E. et al. Ethanol Extract of *Oldenlandia diffusa* Herba Attenuates Scopolamine-Induced Cognitive Impairments in Mice via Activation of BDNF, P-CREB and Inhibition of Acetylcholinesterase

**DOI:** 10.3390/ijms19040996

**Published:** 2018-03-27

**Authors:** Jung Eun Lee, Hyo-Sook Song, Moon Nyeo Park, Sung-Hoon Kim, Bum-Sang Shim, Bonglee Kim

**Affiliations:** 1 Department of Pathology, College of Korean Medicine, Graduate School, Kyung Hee University, 1 Hoegi-dong, Dongdaemun-gu, Seoul 130-701, Korea; jungeunlee@khu.ac.kr (J.E.L.); mnpark@khu.ac.kr (M.N.P.); sungkim7@khu.ac.kr (S.-H.K.); 2 Department of Science in Korean Medicine, College of Korean Medicine, Graduate School, Kyung Hee University, 1 Hoegi-dong, Dongdaemun-gu, Seoul 130-701, Korea; shs331@khu.ac.kr

The authors wish to make the following corrections to this paper [1].

In the Acknowledgments section, we change: “This work was supported by the Basic Science Research Program through the National Research Foundation of Korea (NRF) grant funded by the Ministry of Education (20171825) & Ministry of Science, ICT & Future Planning (20171825)” to “This research was supported by the Basic Science Research Program through the National Research Foundation of Korea (NRF) funded by the Ministry of Education (NRF-2016R1D1A1B03933656)”.

The changes do not affect the scientific results. The authors apologize for any inconvenience this change may cause.
